# Unilateral versus bilateral pedicle screw fixation of minimally invasive transforaminal lumbar interbody fusion (MIS-TLIF): a meta-analysis of randomized controlled trials

**DOI:** 10.1186/1471-2482-14-87

**Published:** 2014-11-06

**Authors:** Liang Wang, Yipeng Wang, Zhengyao Li, Bin Yu, Ye Li

**Affiliations:** Department of Orthopedic Surgery, Peking Union Medical College Hospital, Chinese Academy of Medical Sciences and Peking Union Medical College, 1 Shuaifuyuan Hutong, Beijing, 100730 China

**Keywords:** Transforaminal lumbar interbody fusion (TLIF), Minimally invasive, Unilateral, Bilateral, Pedicle screw fixation

## Abstract

**Background:**

A few studies focused on unilateral or bilateral pedicle screw (PS) fixation of minimally invasive transforaminal lumbar interbody fusion (MIS-TLIF) to treat lumbar degenerative diseases have been published. There is still debate over whether one method is superior to another. A systematic review and meta-analysis of randomized controlled trials (RCT) was performed to compare the efficacy of the two methods.

**Methods:**

We searched the established electronic literature databases of MEDLINE, EMBASE, Web of Science and the Cochrane Central Register of Controlled Trials databases for RCTs comparing the unilateral with bilateral pedicle screw fixation of MIS-TLIF. Pooled mean differences (MD) and odds ratios (OR) and with 95% CIs were calculated for the outcomes.

**Results:**

Three RCTs were identified and analyzed. The results showed that there is no significant difference between the two methods in terms of postoperative VAS-BP score (WMD = -0.09; 95% CI: -0.69 to 0.51; P =0.78), ODI (WMD, -0.09; 95% CI -5.85 to 5.67; P =0.98), fusion rate (OR = 2.99; 95% CI 0.55 to 16.38; P = 0.21) or complication rate (OR = 1.61, 95% CI: 0.49 to 5.37; P =0.43). Unilateral pedicle screw fixation was associated with less blood loss (WMD = -87.83; 95% CI: -160.70 to -14.96; P =0.02).

**Conclusions:**

The existing evidence indicate that no superiority exists between the two fixation methods of MIS-TLIF in terms of functional outcome, fusion rate and complication rate, in spite of that unilateral pedicle screw fixation can achieve less blood loss than bilateral fixation.

## Background

Recently, with the progression of modern instrumentations, the minimally invasive transforaminal lumbar interbody fusion (MIS-TLIF) procedure was adopted in clinical to treat the lumbar degenerative diseases. This minimally invasive approach can be performed using several techniques including a mini-open, endoscopic, or percutaneous. For example, the Sextant system (Medtronic, USA) uses a minimally invasive tubular retractor system where the pedicle screws are percutaneously inserted. The minimally invasive surgery causes lesser soft tissue dissection and allows for early recovery and rehabilitation of the patient. It could lead to less surgical time, blood loss and postoperative pain [1]. Many researches and systematic reviews have indicated that MIS-TLIF is a secure and efficient surgical method and achieve excellent clinical results [2–4].

Generally, bilateral pedicle screw (PS) fixation for MIS-TLIF is accepted as standard procedure, which provides rigid fixation and biomechanical and clinical advantages [5]. However, some studies have shown that greater number of implants and rigid fixation can cause more clinically adverse effects, including reducing fusion rate and adjacent segment degeneration [6, 7]. So, unilateral pedicle screw fixation was put forward to improve these clinical adverse effects. Recent technological advances in spinal instrumentation have promoted the use of unilateral pedicle screw fixation procedure in the MIS-TLIF [8].

Although previous clinical trails reported that unilateral pedicle screw fixation for open or mini-open TLIF acquired similar clinical and fusion results as those of bilateral fixation [9–11]. The effectiveness of unilateral fixation in MIS-TLIF still remains controversial. Thus, the goal of this study is to evaluate the effectiveness of unilateral pedicle screw fixation of MIS-TLIF compared with bilateral fixation and to demonstrate which method is better for treating patients with lumbar degenerative diseases.

## Methods

The study was conducted following the Preferred Reporting Items for Systematic Reviews and Meta-analyses (PRISMA) [12].

### Literature search

Relevant randomized controlled trials (RCTs) were identified by searching MEDLINE, EMBASE, Web of Science and the Cochrane Central Register of Controlled Trials databases. Retrieval time was from the time when databases were built to Dec 2013. We used the following search terms in different combinations as MeSH (Medical Subject Heading) terms and as text words: lumbar degenerative disease, lumbar degenerative disorder, transforaminal lumbar interbody fusion (TLIF), unilateral and bilateral pedicle screw. No linguistic restriction was imposed on the search as recommended by the Cochrane Back Review Group editorial board [13]. To identify other relevant studies, we manually scanned reference lists from identified trials and review articles. Two investigators independently reviewed all subjects, abstracts, and the full text of articles that were potentially eligible based on abstract review. The eligible trials were then selected according to the study eligibility criteria.

### Study eligibility criteria

We systematically reviewed published studies according to the following criteria: (1) randomized, controlled trials; (2) subjects who had undergone one-level MIS-TLIF for lumbar degenerative disorders; (3) the interventions were unilateral and bilateral pedicle screw fixation; (4) the study reported at least one desirable outcome. All potential studies selected by the search strategy were independently reviewed by two investigators for inclusion into the final analysis. Inconsistencies were resolved through discussion until a consensus was reached.

### Data extraction

Two reviewers independently extracted data using a standardized form. Data were collected based on following categories where available. (1) Basic characteristics, including publication year, study design, age, sex, enrolled number, follow-up time and vertebral level treated. (2) Primary outcome is functional outcomes, including visual analgesic score for back pain (VAS-BP) score and Oswestry Disability Index (ODI). (4) Secondary outcomes, consisting of fusion rate, complication rate and estimated blood loss. Any disagreement between the reviewers was resolved by discussion.

### Methodological quality assessment

The study methodological assessment was conducted using the modified Jadad scale [14]. This is an eight item scale designed to assess randomization, blinding, withdrawals and dropouts, inclusion and exclusion criteria, adverse effects and statistical analysis (Table 1). The score for each article could range from 0 (lowest quality) to 8 (highest quality). Scores of 4-8 denote good to excellent quality and 0-3 poor to low quality. Critical appraisal was conducted by one viewer and was verified by another.

**Table 1:** **Modified Jadad score with eight items**

Items assessed	Choi [15]	Xiaolong [16]	Dahdaleh [17]
Was the study described as randomized?	Yes	Yes	Yes
Was the method of randomization appropriate?	Not described	Yes	Not described
Was the study described as blinded?	No	No	No
Was the method of blinding appropriate?	Not described	Not described	Not described
Was there a description of withdrawals and dropouts?	Yes	No	Yes
Was there a clear description of the inclusion/exclusion criteria?	Yes	Yes	Yes
Was the method used to assess adverse effects described?	Yes	Yes	Yes
Was the method of statistical analysis described?	Yes	Yes	Yes
Scores	5	5	5

### Statistical analysis

Continuous variable (VAS-BP, ODI, estimated blood loss) was analyzed using the weighted mean differences (WMD) with its 95% confidence interval (CI), whereas dichotomous data (fusion rate, complication rate) were analyzed using the odd ratio (OR) measure and its 95% CI. Moreover, heterogeneity across trials was evaluated with I^2^ statistic, which defined as I^2^ > 50%. If heterogeneity existed, a random-effect model was used to assess the overall estimate. Otherwise, a fixed-effect model was chosen. All tests were two-tailed and a P value less than 0.05 was regarded as significant in this meta-analysis. Then data were checked and entered into the Review Manager (Version 5.1. Copenhagen: The Nordic Cochrane Centre, The Cochrane Collaboration, 2011) database for further analysis.

## Results

### Selected studies and characteristics

**Figure 1:**
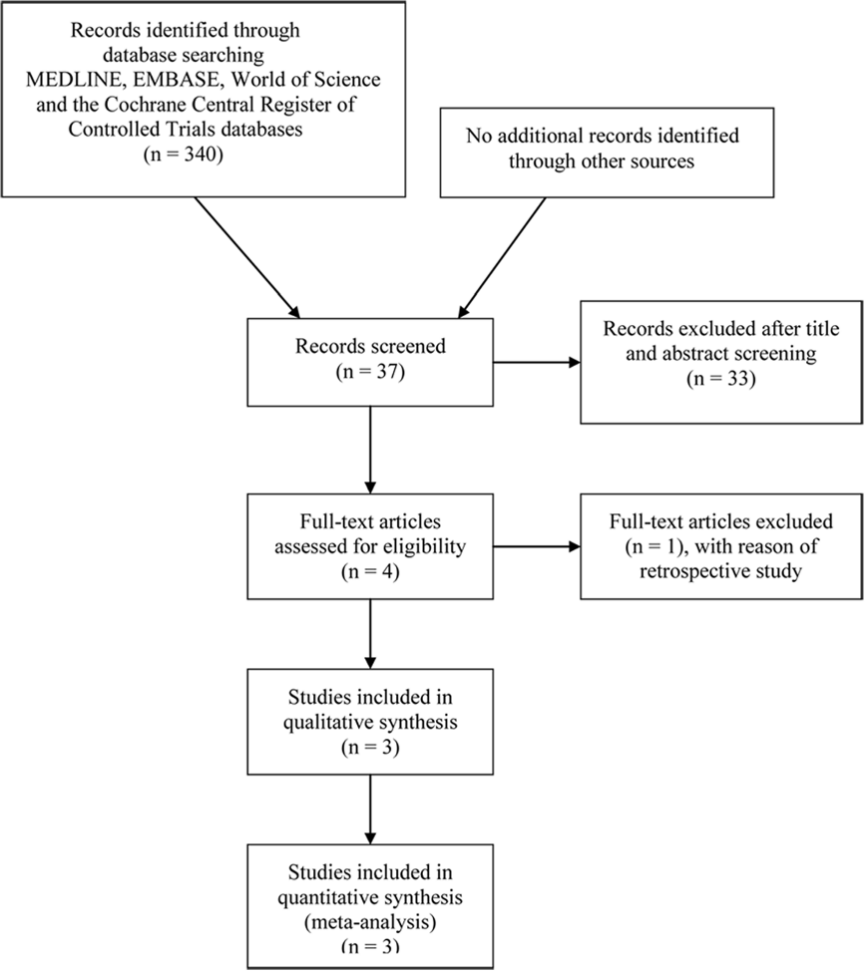
**Flow chart of eligibility selection.**

The details of literature search and selection are displayed in the Figure 1. We identified 340 potentially relevant citations from the initial literature search. After independent review the titles and abstracts of all potential articles, three randomized controlled trials (RCTs) comparing unilateral pedicle screw fixation with bilateral fixation for MIS-TLIF [15–17] were finally identified. All selected studies were in English and all were published in 2013. All three RCT performed their MIS-TLIF with the assistance of Sextant system (Medtronic, USA). The study carried out by Un Yong Choi et al. [15] did not provide data on the Standard Deviation (SD) of constant score. The detailed characteristics of these studies are demonstrated in Tables 2 and 3.

**Table 2:** **Characteristics of included studies**

Study	Number of patients (Female/male)		Age in years		vertebral level treated	Mean follow-up time (month)	
	Unilateral PS	Bilateral PS	Unilateral PS	Bilateral PS		Unilateral PS	Bilateral PS
Choi [15]	26(14/12)	27(18/9)	53.39 ± 14.31	56.22 ± 12.62	L3-S1,one-level	27.52 ± 3.30	28.85 ± 4.37
Xiaolong [16]	31(14/17)	34(18/16)	57.3 ± 11.7	58.9 ± 10.1	L4-S1,one-level	26.6 (18-36)	
Dahdaleh [17]	16(4/12)	20(6/14)	62.2 ± 13.1	57.3 ± 11.2	L3-S1,one-level	11.4 ± 6.1	12.4 ± 7.2

**Table 3:** **Preoperative VAS-BP score and ODI of included studies**

Study	Preoperative VAS-BP score		Preoperative ODI	
	Unilateral PS	Bilateral PS	Unilateral PS	Bilateral PS
Choi [15]	7.6	7.7	27.8	27.9
Xiaolong [16]	6.8 ± 1.6	7.2 ± 2.1	57.56 ± 19.12	51.58 ± 16.38
Dahdaleh [17]	5.7 ± 2.6	5.7 ± 2.5	37.4 ± 9.2	39.2 ± 12

### Methodological quality assessment

The scores of three RCTs [15–17] are shown in Table 1, indicating that all studies achieved high quality by the current rating system. The three RCTs all scored 5, but the main problem reflected in all studies was the nonuse of blinding method, which might bring about a certain degree of detection bias. Only one trail [16] reported the randomization method, which used randomization of computer-generated number list.

### Meta-analysis results

#### Functional outcomes

**Figure 2:**
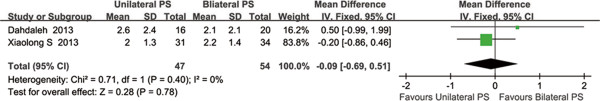
**Forest plot: weighted mean difference (WMD) in postoperative VAS-BP score and 95% CI for unilateral versus bilateral pedicle screw fixation of MIS-TLIF.** No significant difference was found between the two groups.

**Figure 3:**
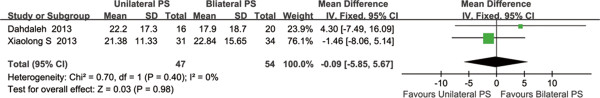
**Forest plot: weighted mean difference (WMD) in postoperative ODI and 95% CI for unilateral versus bilateral pedicle screw fixation of MIS-TLIF.** No significant difference was found between the two groups.

All the three RCTs applied the indexes to evaluate the functional outcomes of unilateral and bilateral pedicle screw fixation for MIS-TLIF. The indexes include VAS-BP score and ODI. However, only two studies [16, 17] provide the mean and SD of the outcome. Statistical analysis was feasible after standardization pooling for comparing functional outcome. Improvement in functional status postoperatively was identified for both interventions, but there was no significant difference between the two intervention groups. For the postoperative VAS-BP score, the pooled analysis showed no difference (WMD = -0.09; 95% CI: -0.69 to 0.51; P =0.78; fixed effect model) with no heterogeneity (P =0.40, I^2^ = 0%) between the two groups (Figure 2). In addition, postoperative functional performance was also assessed using the ODI questionnaire. Analysis indicated no difference (WMD, -0.09; 95% CI -5.85 to 5.67; P =0.98; fixed effect model) with no heterogeneity (P =0.40, I^2^ = 0%) between the two groups (Figure 3).

#### Estimated blood loss

**Figure 4:**
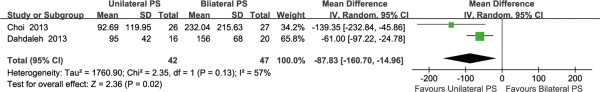
**Forest plot: weighted mean difference (WMD) in estimated blood loss and 95% CI for unilateral versus bilateral pedicle screw fixation of MIS-TLIF.** Lower blood loss in the unilateral group was observed in this forest plot.

Two RCTs [15, 17] reported the estimated blood loss for both unilateral and bilateral fixation of MIS-TLIF procedure. Our pooled results showed that there is significantly less estimated blood loss for the unilateral fixation group than bilateral fixation counterparts (WMD = -87.83; 95% CI: -160.70 to -14.96; P =0.02; random effect model) with the heterogeneity of I^2^ = 57%, P =0.13 (Figure 4).

#### Fusion rate

**Figure 5:**
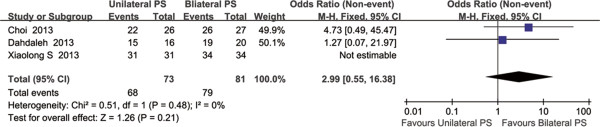
**Forest plot: odds ratio (OR) in fusion rate and 95% CI for unilateral versus bilateral pedicle screw fixation of MIS-TLIF.** No significant difference was found between the two groups.

All the three RCTs reported the fusion rate in regards to their latest follow-up. Fusion was assessed with the use of dynamic flexion-extension radiographs and CT scan. The fusion rate was 93.15% (68/73) in the unilateral fixation group and 97.53% (79/81) in the bilateral fixation group. Although the fusion rate was higher in bilateral group, but there was no significant difference between the two groups (OR = 2.99; 95% CI 0.55 to 16.38; P = 0.21; fixed effect model) with no heterogeneity (P = 0.48, I^2^ = 0%) (Figure 5).

#### Complication rate

**Figure 6:**
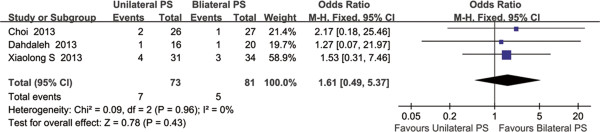
**Forest plot: odds ratio (OR) in complication rate and 95% CI for unilateral versus bilateral pedicle screw fixation of MIS-TLIF.** No significant difference was found between the two groups.

The three RCTs presented the number of case suffering from complications. The complication rate was 9.59% (7/73) in the unilateral fixation group and 6.13% (5/81) in the bilateral fixation group. The pooled data revealed that there was no significant difference in the complication rate (OR = 1.61, 95% CI: 0.49 to 5.37; P =0.43; fixed effect model) with no heterogeneity (P = 0.96, I^2^ = 0%), which reflects the primary harm outcome (Figure 6).

## Discussion

To our knowledge, this is the first quantitative comparative meta-analysis study comparing unilateral and bilateral pedicle screw fixation of MIS-TLIF for the treatment of lumbar degenerative diseases. Ultimately, three RCTs in the literatures were included in our systematic review. In order to assess the efficacy of the two methods, we extracted relative data as much as possible and pooled the outcome whenever possible.

Among various spinal fusion techniques, TLIF has become a popular and established technique since it could reduce the amount of thecal sac and nerve root retraction through the lateral approach to the disc space [18–20]. Although the open TLIF procedure preserves the major portion of the posterior ligament complex with minimal compromise of spinal stability, it also needs to dissect and retract paraspinal muscle, which could cause muscle denervation, atrophy and consequently postoperative low back pain [21]. Then, with the advent of modern image guidance and sophisticated instrumentations, the MIS-TLIF was introduced by Foley et al. for the first time in 2002 [22]. Since then, it has become an increasing popular technique and has been proven advantageous to traditional open surgery in terms of damage upon spinal soft tissues [20, 23, 24].

As for the MIS-TLIF procedure, the need for unilateral or bilateral pedicle screw fixation is a controversial topic. Several studies showed that the unilateral fixation for TLIF acquires good outcomes, including open and minimally invasive procedures [1, 9]. According to the original technique, transforaminal approach should be unilateral, preserving contralateral laminae and articular facets. It requires unilateral total facetetomy and may cause iatrogenic instability. The unilateral pedicle screw fixation for the MIS-TLIF may cause insufficiently stable construct and result in a higher incidence of instrumentation failure and nonunion. Previous biomechanical studies showed that unilateral fixation after TLIF provided less rotational stability and stiffness than bilateral pedicle screw fixation [25–27]. Despite data suggesting the inferiority of unilateral pedicle screw fixation after TLIF in biomechanical studies, some studies reported that unilateral pedicle screw fixation for TLIF acquired similar clinical and fusion results as those of bilateral fixation [9–11]. However, these trials used a conventional pedicle screw system or mini-open TLIF instead of a percutaneous pedicle screw and MIS-TLIF procedure. Recently, some articles were published to compare the unilateral with bilateral fixation of MIS-TLIF. So, the present meta-analysis of randomized controlled trials was designed to evaluate whether one method is superior to another one.

In the systematic review, we took the outcome of postoperative VAS-BP score and ODI for functional assessment. Because one trial only provided the mean score, only two RCTs were included in the meta-analysis of clinical efficacy. As for VAS-BP score, overall outcome showed that there was no significant difference across these two interventions. A similar trend was also found when assessing for ODI. The meta-analysis reveals that unilateral pedicle screw fixation for MIS-TLIF can acquire equal clinical functional improvement as the bilateral fixation.

The main function of the pedicle screw fixation is to stabilize the spine and promote fusion, so the fusion rate is the most important outcome to consider. The meta-analysis found that unilateral pedicle screw fixation of MIS-TLIF was associated with a lower fusion rate than bilateral fixation counterparts with OR of 0.33, but there is no significant difference. Prior research found that unilateral pedicle screw fixation could reduce the fusion rate due to the less rotational stability and stiffness [28]. As for traditional mini-open TLIF procedure, Suk [29] and Xue [11] both reported the relative lower fusion rate of unilateral fixation than bilateral group. However, a meta-analysis of unilateral versus bilateral pedicle screw fixation for lumbar interbody fusion revealed that both unilateral and bilateral fixation could achieve satisfactory fusion rate with no statistical differences [30].

In addition, Choi reported that postoperative scoliotic change occurred highly in unilateral than bilateral group (23.1% versus 3.7%) [15]. During the unilateral pedicle screw fixation of MIS-TLIF, unilateral compression force may cause spinal asymmetric and postoperative scoliotic change. In their research, the patients with scoliosis did not have clinical symptoms. In Shen’s study [16], there were no significant differences between the two groups in relation to lumbar scoliosis angle and segmental scoliosis angle.

As for the complication, the three RCTs all reported the details. The meta-analysis revealed that there is no significant difference between the two fixation groups. In addition, the previous mentioned meta-analysis showed that the two methods had no significant difference in the complication rate [30]. For complications related the fusion surgery, hardware related complications often cause serious effects. In unilateral PS group of MIS-TLIF, one patient suffered from root irritation due to the violation of pedicle cortex by screw. Two patients experienced cage migration and one underwent reoperation. For bilateral PS fixation, one patient suffered from malposition of pedicle screw and reoperation. One patient underwent revision surgery due to upper segment disc herniation. In addition, some minor complications were reported, including superficial wound infection, urinary tract infection and dural tear, which were all treated conservatively.

In addition, we did the meta-analysis of estimated blood loss for the two fixation methods. The unilateral pedicle screw fixation of MIS-TLIF had significantly less blood loss than bilateral fixation group. The unilateral fixation usually combines with unilateral decompression, which has less damage in the surrounding tissue and uses less pedicle screws. So, the blood loss is significantly superior to bilateral procedure.

There are several potential limitations in this meta-analysis. Firstly, one prominent drawback pertinent to this study is that only three RCTs with 154 subjects were included in this meta-analysis. The results of pooled analysis might therefore be accompanied with bias. Secondly, only few functional and radiological outcome measures were examined in the present study. More parameters, including objective and subjective measures, should be recorded to evaluate the clinical and radiological efficacy. Thirdly, the included studies were short or medium-term research. Whether both surgical methods can provide equal efficacy in long-term follow-up remain unknown. Long-term follow-up efficacy of the two methods should be evaluated.

## Conclusions

In conclusion, our meta-analysis indicates that unilateral pedicle screw fixation of MIS-TLIF acquired similar functional efficacy as bilateral fixation. In addition, there is no significant difference of interbody fusion rate and complication rate between the two methods. The unilateral pedicle screw fixation leads to less blood loss than bilateral fixation. Considering the limitations of included studies, only a limited recommendation can be made based on current data. And large samples, well designed randomized controlled clinical trials that incorporates the evaluation of clinical and radiological outcomes are required to assess the two fixation procedures in the future.

## Abbreviations

WMD: Weighted mean difference
OR: Odds ratio
SD: Standard Deviation
CI: Confidence interval
RCT: Randomized controlled trial
PS: Pedicle screw
TLIF: Transforaminal lumbar interbody fusion
MIS-TLIF: Minimally invasive transforaminal lumbar interbody fusion
VAS-BP: Visual analgesic score for back pain
ODI: Oswestry Disability Index.

## References

[1] Beringer WF, Mobasser JP (2006). Unilateral pedicle screw instrumentation for minimally invasive transforaminal lumbar interbody fusion. Neurosurg Focus.

[2] Wu RH, Fraser JF, Hartl R (2010). Minimal access versus open transforaminal lumbar interbody fusion: meta-analysis of fusion rates. Spine (Phila Pa 1976).

[3] Tian NF, Wu YS, Zhang XL, Xu HZ, Chi YL, Mao FM (2013). Minimally invasive versus open transforaminal lumbar interbody fusion: a meta-analysis based on the current evidence. Eur Spine J.

[4] Sun ZJ, Li WJ, Zhao Y, Qiu GX (2013). Comparing minimally invasive and open transforaminal lumbar interbody fusion for treatment of degenerative lumbar disease: a meta-analysis. Chin Med J (Engl).

[5] Sim HB, Murovic JA, Cho BY, Lim TJ, Park J (2010). Biomechanical comparison of single-level posterior versus transforaminal lumbar interbody fusions with bilateral pedicle screw fixation: segmental stability and the effects on adjacent motion segments. J Neurosurg Spine.

[6] Lee CS, Hwang CJ, Lee SW, Ahn YJ, Kim YT, Lee DH, Lee MY (2009). Risk factors for adjacent segment disease after lumbar fusion. Eur Spine J.

[7] Park P, Garton HJ, Gala VC, Hoff JT, McGillicuddy JE (2004). Adjacent segment disease after lumbar or lumbosacral fusion: review of the literature. Spine (Phila Pa 1976).

[8] Schizas C, Tzinieris N, Tsiridis E, Kosmopoulos V (2009). Minimally invasive versus open transforaminal lumbar interbody fusion: evaluating initial experience. Int Orthop.

[9] Deutsch H, Musacchio MJ (2006). Minimally invasive transforaminal lumbar interbody fusion with unilateral pedicle screw fixation. Neurosurg Focus.

[10] Tuttle J, Shakir A, Choudhri HF (2006). Paramedian approach for transforaminal lumbar interbody fusion with unilateral pedicle screw fixation. Technical note and preliminary report on 47 cases. Neurosurg Focus.

[11] Xue H, Tu Y, Cai M (2012). Comparison of unilateral versus bilateral instrumented transforaminal lumbar interbody fusion in degenerative lumbar diseases. Spine J.

[12] Liberati A, Altman DG, Tetzlaff J, Mulrow C, Gotzsche PC, Ioannidis JP, Clarke M, Devereaux PJ, Kleijnen J, Moher D (2009). The PRISMA statement for reporting systematic reviews and meta-analyses of studies that evaluate health care interventions: explanation and elaboration. PLoS Med.

[13] Furlan AD, Pennick V, Bombardier C, van Tulder M (2009). 2009 updated method guidelines for systematic reviews in the Cochrane Back Review Group. Spine (Phila Pa 1976).

[14] Oremus M, Wolfson C, Perrault A, Demers L, Momoli F, Moride Y (2001). Interrater reliability of the modified Jadad quality scale for systematic reviews of Alzheimer’s disease drug trials. Dement Geriatr Cogn Disord.

[15] Choi UY, Park JY, Kim KH, Kuh SU, Chin DK, Kim KS, Cho YE (2013). Unilateral versus bilateral percutaneous pedicle screw fixation in minimally invasive transforaminal lumbar interbody fusion. Neurosurg Focus.

[16] Xiaolong S, Lei W, Hailong Z, Xin G, Guangfei G, Shisheng H (2013). Radiographic analysis of one-level Minimally Invasive Transforaminal Lumbar Interbody Fusion (MI-TLIF) with unilateral pedicle screw fixation for lumbar degenerative diseases. J Spinal Disord Tech.

[17] Dahdaleh NS, Nixon AT, Lawton CD, Wong AP, Smith ZA, Fessler RG (2013). Outcome following unilateral versus bilateral instrumentation in patients undergoing minimally invasive transforaminal lumbar interbody fusion: a single-center randomized prospective study. Neurosurg Focus.

[18] Moskowitz A (2002). Transforaminal lumbar interbody fusion. Orthop Clin North Am.

[19] Hackenberg L, Halm H, Bullmann V, Vieth V, Schneider M, Liljenqvist U (2005). Transforaminal lumbar interbody fusion: a safe technique with satisfactory three to five year results. Eur Spine J.

[20] Schwender JD, Holly LT, Rouben DP, Foley KT (2005). Minimally invasive transforaminal lumbar interbody fusion (TLIF): technical feasibility and initial results. J Spinal Disord Tech.

[21] Tsahtsarlis A, Wood M (2012). Minimally invasive transforaminal lumber interbody fusion and degenerative lumbar spine disease. Eur Spine J.

[22] Foley KT, Lefkowitz MA (2002). Advances in minimally invasive spine surgery. Clin Neurosurg.

[23] Mummaneni PV, Rodts GE (2005). The mini-open transforaminal lumbar interbody fusion. Neurosurgery.

[24] Scheufler KM, Dohmen H, Vougioukas VI (2007). Percutaneous transforaminal lumbar interbody fusion for the treatment of degenerative lumbar instability. Neurosurgery.

[25] Harris BM, Hilibrand AS, Savas PE, Pellegrino A, Vaccaro AR, Siegler S, Albert TJ (2004). Transforaminal lumbar interbody fusion: the effect of various instrumentation techniques on the flexibility of the lumbar spine. Spine (Phila Pa 1976).

[26] Schleicher P, Beth P, Ottenbacher A, Pflugmacher R, Scholz M, Schnake KJ, Haas NP, Kandziora F (2008). Biomechanical evaluation of different asymmetrical posterior stabilization methods for minimally invasive transforaminal lumbar interbody fusion. J Neurosurg Spine.

[27] Slucky AV, Brodke DS, Bachus KN, Droge JA, Braun JT (2006). Less invasive posterior fixation method following transforaminal lumbar interbody fusion: a biomechanical analysis. Spine J.

[28] Yucesoy K, Yuksel KZ, Baek S, Sonntag VK, Crawford NR (2008). Biomechanics of unilateral compared with bilateral lumbar pedicle screw fixation for stabilization of unilateral vertebral disease. J Neurosurg Spine.

[29] Suk KS, Lee HM, Kim NH, Ha JW (2000). Unilateral versus bilateral pedicle screw fixation in lumbar spinal fusion. Spine (Phila Pa 1976).

[30] Yuan C, Chen K, Zhang H, Zhang H, He S (2014). Unilateral versus bilateral pedicle screw fixation in lumbar interbody fusion: a meta-analysis of complication and fusion rate. Clin Neurol Neurosurg.

[CR31] The pre-publication history for this paper can be accessed here:http://www.biomedcentral.com/1471-2482/14/87/prepub

